# Self-Assembly and Antimicrobial Activity of Lipopeptides Containing Lysine-Rich Tripeptides

**DOI:** 10.1021/acs.biomac.3c01184

**Published:** 2024-01-11

**Authors:** Anindyasundar Adak, Valeria Castelletto, Ana de Sousa, Kimon-Andreas Karatzas, Callum Wilkinson, Nikul Khunti, Jani Seitsonen, Ian W. Hamley

**Affiliations:** † School of Chemistry, Pharmacy and Food Biosciences, University of Reading, Whiteknights, Reading RG6 6AH, U.K.; ‡ Diamond Light Source, Harwell Science and Innovation Campus, Chilton, Didcot OX11 0DE, U.K.; § Nanomicroscopy Center, Aalto University, Puumiehenkuja 2, FIN-02150 Espoo, Finland

## Abstract

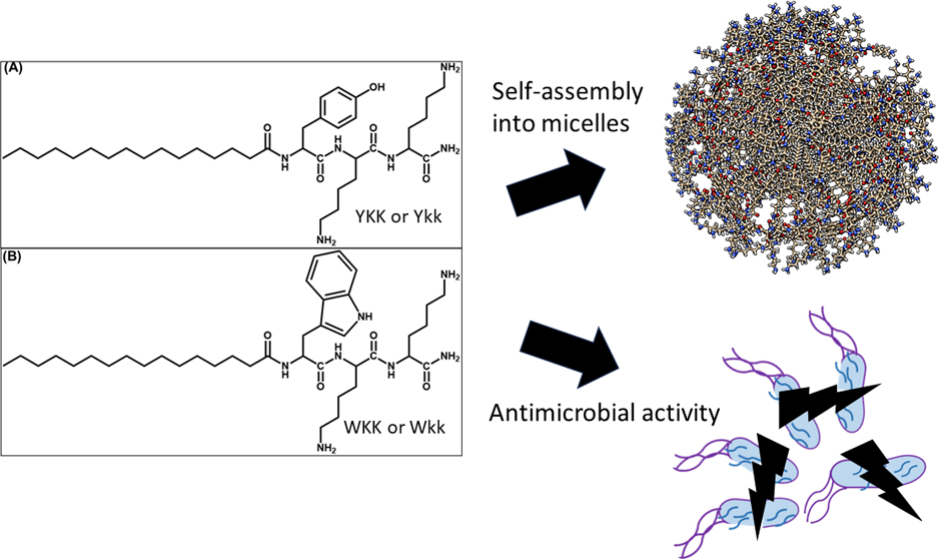

The conformation and self-assembly of two pairs of model lipidated tripeptides in aqueous solution are probed using a combination of spectroscopic methods along with cryogenic-transmission electron microscopy (cryo-TEM) and small-angle X-ray scattering (SAXS). The palmitoylated lipopeptides comprise C_16_-YKK or C_16_-WKK (with two l-lysine residues) or their respective derivatives containing d-lysine (k), i.e., C_16_-Ykk and C_16_-Wkk. All four molecules self-assemble into spherical micelles which show structure factor effects in SAXS profiles due to intermicellar packing in aqueous solution. Consistent with micellar structures, the tripeptides in the coronas have a largely unordered conformation, as probed using spectroscopic methods. The molecules are found to have good cytocompatibility with fibroblasts at sufficiently low concentrations, although some loss of cell viability is noted at the highest concentrations examined (above the critical aggregation concentration of the lipopeptides, determined from fluorescence dye probe measurements). Preliminary tests also showed antimicrobial activity against both Gram-negative and Gram-positive bacteria.

## Introduction

Peptide amphiphiles (PAs) are a remarkable class of molecules built from biomimetic elements that can be designed to produce self-assembled nanostructures with an impressive range of demonstrated bioactivities.^1,2^ They can be designed and constructed in various ways. One class of PA is surfactant-like peptides (SLPs) in which the peptide sequence has an amphiphilic character due to sequences of hydrophobic and hydrophilic amino acids. Another class of PAs is formed by the covalent conjugation of lipid chains or aromatic moieties to peptides.^3−5^ Generally, the PAs are constructed by the attachment of a lipid chain or aromatic moiety at the N-terminal of the desired peptide sequences. In the self-assembly process, the lipid chain or aromatic moiety will be at the core of the nanostructure because of its hydrophobic character, and a hydrophilic or charged C-terminal peptide will form the exterior corona of the nanostructure and act as a polar headgroup.^6,7^ Depending on the peptide sequence, length, and properties of the hydrophobic moiety, different self-assembled nanostructures are formed.^8,9^ The most commonly reported are fibers (or nanofibers, also known as fibrils or nanofibrils), these being favored by sequences that drive β-sheet conformation. However, other morphologies including nanotubes, nanorings, and nanosheets are also observed. Lipopeptide micelles (including wormlike micelles and spherical micelles) have also been observed for conjugates containing α-helical peptides^10,11^ or short peptide sequences,^12−21^ conjugates containing intrinsically disordered peptides,^22^ or even cyclic lipopeptides.^23−27^

Antimicrobial peptides (AMPs) are currently attracting attention because of the antibiotic resistance of traditional antibiotics. One class of AMPs is endogenous molecules, which can be found in the innate immune system of living organisms.^28^ Another type of AMP contains cationic residues that interact with anionic or zwitterionic bacterial cell membranes, leading to lysis and the leakage of ions, nutrients, and cytoplasmic components, and ultimately killing the bacteria without the threat of bacterial resistance.^28−30^ Based on this mode of action, several AMPs have been designed, developed, and isolated from various organisms.^31^

Among the diverse applications of PAs, they can show useful in vivo AMP activity because their N-terminus is protected by acylation, which improves stability against proteolytic degradation. This can also be enhanced by incorporating nonnatural residues or d-amino acid variants into the peptide sequence.^32^ Among studies reported on lipidated lysine-rich short-peptides, the self-assembled structures of mono- and di-lipidated KKK and KEK peptides have been probed, and the formation of a ribbon-like structure was observed for di-lipid peptide conjugates, but for the mono-lipid peptide conjugate, no dominant self-assembled nanostructure was observed.^33^ The self-assembly of C_16_-KKF and C_16_-KKFF cleaved from the PA C_16_-KKFFVLK led to the formation of spherical micelles.^34^ Later, the antimicrobial activity of C_16_-KKFF in a hybrid material with alginate and graphene oxide was examined, and significant antimicrobial activity specific to the Gram-positive bacterium *Listeria monocytogenes* was noted.^35^ Another recent study reports a very thorough investigation of the self-assembly, antibacterial, and wound-healing properties of lysine-rich lipopeptides incorporating amyloid sequences to drive β-sheet fibril formation.^36^ Laverty and co-workers developed antimicrobial lipopeptides of the form C_n_-OOWW (*n* = 6–16, and O denotes ornithine, the analogue of lysine with one less methyl group in the side chain).^37^ Most closely related to the study here, Makovitzki et al. reported lipopeptides comprising palmitic acid conjugated to cationic di- and tri-peptides containing all l-amino acids or mixed d,l-sequences and observed significant antimicrobial activity, although the conjugates containing d-amino acid did not display any enhanced activity compared to those based on l-amino acids.^38^ Watson et al. reported a comprehensive comparison of cellular uptake and delivery ability of two cell-penetrating peptides P16 and its truncated version P7, where the tripeptide WKK at the C-terminal of both peptides played a crucial role.^39^ Further, this class of amphiphilic molecule has been further developed or biomedical applications by the design of novel conjugates linking hydrophilic peptides and hydrophobic molecules such as drugs.^40,41^

Here, we report on a series of designed lipopeptides bearing minimal cationic sequences to confer the expected antimicrobial properties. The tripeptides contain two lysine residues (as either l- or d-amino acids, K or k, respectively) as well as aromatic residues tyrosine (Y) or tryptophan (W) to modulate amphiphilicity. The self-assembly propensity of all 20^3^ = 8000 native tripeptide sequences has been examined by computer simulation, and it was observed that those tripeptides that included hydrophilic residues along with aromatic residue were more prone to self-assembly propensity.^42^ The results of this study indicate that YKK and WKK are not expected to have a high aggregation tendency. High aggregation propensity is generally favored by sequences with aromatic residues as the second and/or third positions, whereas positively charged or hydrogen-bonding residues occupy the N-terminal position and acidic residues are favored at the C-terminus. We reasoned that conjugation of our tripeptides to N-terminal hexadecyl (C_16_) (palmitoyl) chains would lead to surfactant-like lipopeptides with amphiphilic character.

**Figure 1:**
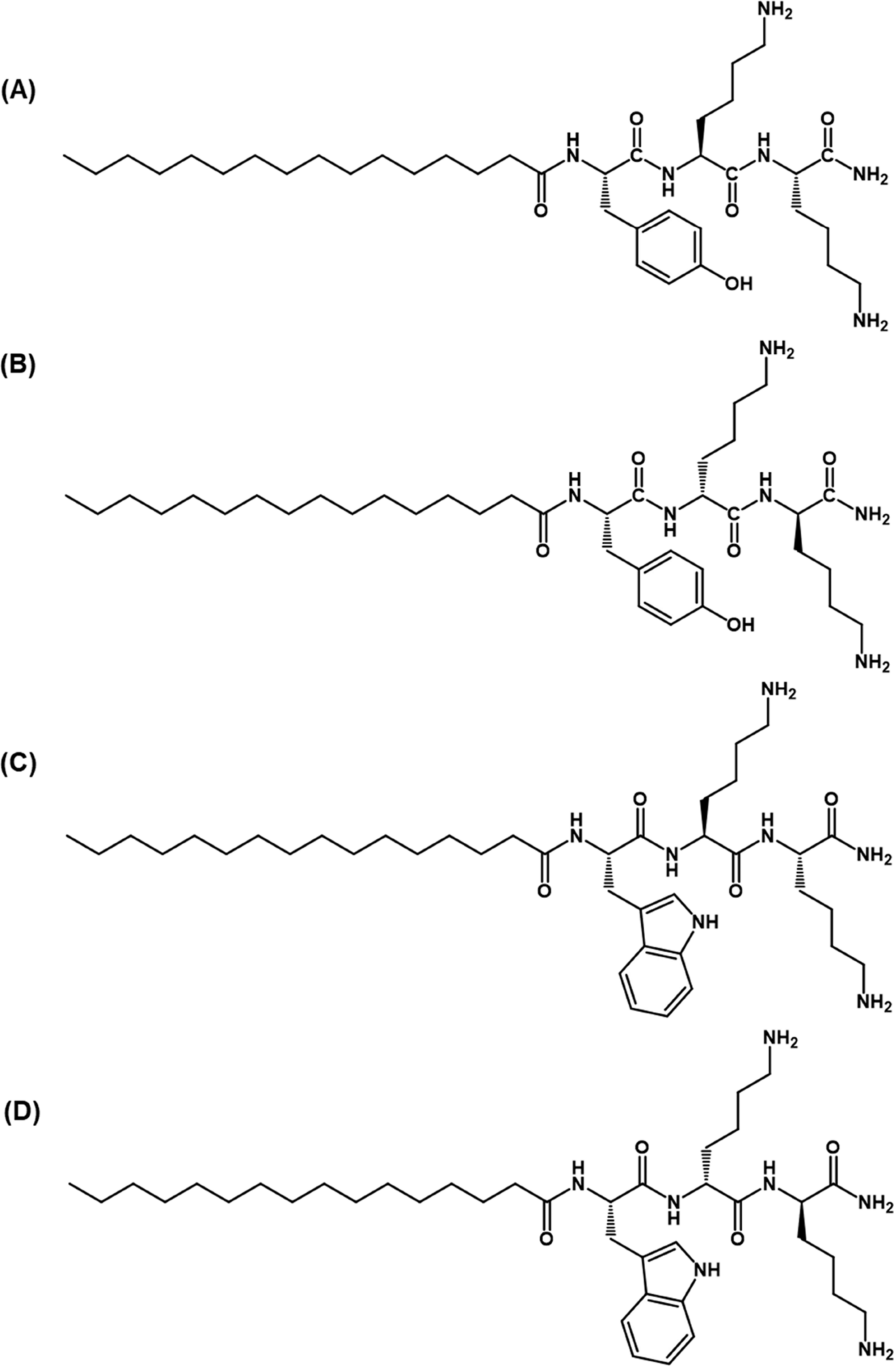
Structures of (A) **P1**, (B) **P1D**, (C) **P2,** and (D) **P2D**. All peptides have an amidated C-terminus.

Herein, we present a study of self-assembly and preliminary bioactivity (cytocompatibility and antimicrobial activity) investigations of the four lipopeptides C_16_-YKK, C_16_-Ykk, C_16_-WKK, and C_16_-Wkk with structures as shown in Figure 1. Here, they are abbreviated as **P1**, **P1D**, **P2**, and **P2D**, respectively. The conformation of the four conjugates was studied in aqueous solution using circular dichroism (CD) and Fourier transform infrared (FTIR) spectroscopies. Critical aggregation concentrations (CACs) were determined from fluorescence probe assays. The mode of self-assembly was determined using cryogenic-transmission electron microscopy (cryo-TEM) and small-angle X-ray scattering (SAXS). These techniques show that all four molecules form spherical micelles above the CAC. Cell viability studies show low cytotoxicity to fibroblasts at sufficiently low concentrations (although some loss of viability was observed for some molecules above the CAC). Assays to estimate minimum inhibitory concentration (MIC) values indicate that the compounds display significant antimicrobial effects against Gram-negative *Escherichia coli*, *Salmonella enterica*, and Gram-positive *Staphylococcus aureus*.

## Experimental Section (Materials and Methods)

### Materials

#### Chemicals

Rink amide resin, Fmoc amino acids, diisopropylethylamine (DIPEA), and O-(1-benzotriazolyl)-1,1,3,3-tetramethyluronium hexafluorophosphate (HBTU), and triisopropylsilane (TIS) were obtained from Sigma-Aldrich. Methanol, trifluoroacetic acid (TFA), piperidine, diethyl ether, phenol, dichloromethane, *N*,*N*′-dimethylformamide (DMF), HPLC grade water, and HPLC grade acetonitrile were purchased from Thermo-Fisher. Lipopeptides were purified using an Agilent 1200 HPLC with a Supelco C-18 column (Zorbax ODS HPLC column 15 × 4.6 mm, 5 μm) using a gradient of acetonitrile/water at a flow rate of 1.2 min/ml, for a runtime of 30 min.

### Synthesis of Lipopeptides

Solid-phase peptide synthesis was performed using Rink amide resin as the solid support. Piperidine (20% v/v) in DMF was used for the deprotection of the Fmoc [fluorenylmethoxycarbonyl] group. For each coupling step, Fmoc amino acids (5 equiv), DIPEA (12 equiv), and HBTU (5 equiv) were used by dissolving in DMF. The reactions for the coupling step and deprotection step were performed by purging nitrogen gas for 6 h and 30 min, respectively. The free carboxylic group of palmitic acid was coupled at the N-terminal of the synthesized peptide using HBTU and DIPEA in DMF for 6 h. The lipopeptide was cleaved from the resin with a cocktail mixture containing TFA, TIS, and H_2_O [96:2.0:2.0 (v/v/v)], at room temperature for 4 h. Next, TFA was evaporated with nitrogen gas to a minimum volume, and ice-cold diethyl ether was added to yield a white precipitate. The precipitate was centrifuged, lyophilized, and purified by reversed-phase high-performance liquid chromatography. Each synthesized final molecule was characterized by HPLC (SI Figures S1, S3, S5, and S7) and ESI-MS (SI Figures S2, S4, S6, and S8). The mass values obtained agree with the expected molar masses **P1** and **P1D**: M_theo_ = 674.51 g mol^–1^ and **P2** and **P2D**: M_theo_ = 697.53 g mol^–1^. The observed values are **P1**: M_obs_ = 675.51 g mol^–1^, **P1D**: M_obs_ = 675.52 g mol^–1^, **P2**: M_obs_ = 698.53 g mol^–1^, and **P2D**: M_obs_ = 698.53 g mol^–1^. The purities of the lipopeptides analyzed by HPLC are as follows: **P1** = 99.62%, **P1D** = 99.70%, **P2** = 99.49%, and **P2D** = 96.69% (SI Figures S1–S3, S5, and S7).

### Sample Preparation

Measured amounts of lipopeptide were dissolved in water to obtain samples with defined concentrations in wt %, and their pH was measured using a Mettler-Toledo FiveEasy pH meter with a Sigma-Aldrich micro-pH combination electrode (glass body), and the pH was found to be approximately 4.6 for the samples studied here.

### CD Spectroscopy

The CD spectra of the lipopeptides were obtained by using a Chirascan spectropolarimeter (Applied Photophysics, Leatherhead, UK) equipped with a thermal controller. The samples were placed in 0.1 mm quartz cells. All the spectra were recorded 3 times in the range of 280 to 180 nm, with 0.5 nm step, 1 nm bandwidth, and 1 s collection time per step. Here, each CD spectrum of the sample was background subtracted using the spectrum of water.

### FTIR Spectroscopy

The FTIR spectra of the lipopeptides were recorded using a Thermo-Scientific Nicolet iS5 instrument with a deuterated triglycine sulfate (DTGS) detector, with a Specac Pearl liquid cell containing CaF_2_ plates, where the sample was fixed. A total of 128 scans for each sample were recorded over the range of 900–4000 cm^–1^.

### Fluorescence Spectroscopy

Fluorescence experiments were carried out using a Varian Cary Eclipse spectrofluorometer in 4 mm inner-width quartz cuvettes. Excitation and emission bandwidths of 2.5 nm were used as the experimental settings. The temperature was set at 25 °C for all of the experiments. The CAC value for all of the lipopeptides was assessed by fluorescence experiments with 8-anilo-1-naphthalenesulfonic acid (ANS). ANS is a suitable fluorophore to determine the CAC value, due to changes in local hydrophobicity.^43^ For this assay, various concentrations of samples were prepared in 2 × 10^–3^ wt % ANS solution. Fluorescence spectra were recorded from 400 to 670 nm with λ_ex_ = 356 nm.

To determine the CAC value of individual lipopeptides, the results were plotted as *I*/*I*_0_ versus log(c/wt %), where *I* stands for the maximum fluorescence intensity of ANS containing the various concentrations of samples and *I*_0_ stands for the intensity for the control, i.e., ANS solution without lipopeptide.

### Cryogenic-Transmission Electron Microscopy

Imaging was carried out using a field emission cryoelectron microscope (JEOL JEM-3200FSC), operating at 200 kV. Images were taken in bright field mode and using zero loss energy filtering (omega type) with a slit width of 20 eV. Micrographs were recorded using a Gatan Ultrascan 4000 CCD camera. The specimen temperature was maintained at −187 °C during the imaging. Vitrified specimens were prepared using an automated FEI Vitrobot device using Quantifoil 3.5/1 holey carbon copper grids with a hole size of 3.5 μm. Just prior to use, grids were plasma cleaned using a Gatan Solarus 9500 plasma cleaner and then transferred into the environmental chamber of an FEI Vitrobot at room temperature and 100% humidity. Thereafter, 3 μL of sample solution was applied on the grid and was blotted twice for 5 s and then vitrified in a 1/1 mixture of liquid ethane and propane at a temperature of −180 °C. The grids with vitrified sample solution were maintained at liquid nitrogen temperature and then cryo-transferred to the microscope.

### Small-Angle X-ray Scattering

SAXS experiments were performed on beamline B21 at Diamond (Didcot, UK). The sample solutions were loaded into the 96-well plate of an EMBL BioSAXS robot and then injected via an automated sample exchanger into a quartz capillary (1.8 mm internal diameter) with the X-ray beam. The quartz capillary was enclosed in a vacuum chamber to avoid parasitic scattering. After the sample was injected into the capillary and reached the X-ray beam, the flow was stopped during SAXS data acquisition. Beamline B21 operates with a fixed camera length (3.9 m) and fixed energy (12.4 keV). The images were captured by using a PILATUS 2 M detector. Data processing was performed by using the dedicated beamline software ScÅtter.

### Cell Lines

L929 murine fibroblast cell lines (ECACC General Cell Collection) were grown in Dulbecco’s modified Eagle’s medium (DMEM) supplemented with 10% fetal bovine serum (FBS), 20 mM HEPES, and 1% GlutaMAX. The cells were maintained at pH 7.4, 37 °C, and 5% CO_2_ in 25 cm^2^ cell culture flasks.

### Cytotoxicity Assays

For the cytotoxicity assay, approximately 6000 cells per well were seeded in 96-well plates. The medium was exchanged after 24 h by adding solutions of different concentrations of the lipopeptides ranging from 0.05 to 0.0001 wt %. After 48 h of treatment, cell viability was assessed using an MTT [3-(4, 5-dimethylthiazolyl-2)-2, 5-diphenyltetrazolium bromide] assay. Here, the culture medium was removed, followed by the addition of a solution of MTT in DMEM medium at a concentration of 0.5 mg/mL. The plate was incubated for 4 h at 37 °C. Next, to dissolve the formazan crystals, dimethyl sulfoxide was added. Thereafter, using an automatic plate reader, the absorbance was measured at 560 nm. Cell survival was expressed as a percentage of viable cells in the presence of peptides compared with control cells grown in their absence. The assay was repeated three times, and the results were averaged. Statistical significance was tested using multiple Student’s *t*-tests.

### Antibacterial Susceptibility Assay

Antibacterial susceptibility testing was performed using a broth microdilution assay, against both Gram-negative and Gram-positive bacteria, as previously described by Kourmentza et al.^44^ The target strains used were *E. coli* K-12, *S. enterica* NCTC 5188, and *S. aureus* NCDO 949. The test compounds were dissolved in water, filter-sterilized, and further diluted in Mueller-Hinton broth (MHB) which was used as the growth medium for the tests. 2-fold dilutions were performed, and 100 μL of each dilution was transferred to each well of 96-well plates. The final test concentration range of the compounds was 1.95–1000 μg/mL. Additionally, the 96-well plates contained wells with 200 μL of growth medium that served as sterility controls and wells filled with 100 μL of growth medium and 100 μL of inoculum that served as growth controls. The remaining wells were inoculated with 100 μL of a bacterial culture. The bacterial suspensions were prepared from overnight cultures in MHB. The bacterial density was adjusted to 0.5 McFarland (standard), and the suspensions were subsequently diluted 100 times using the growth medium. The 96-well plates were incubated at 37 °C for 24 h and the OD at 620 nm of the wells was assessed for growth. The MIC was defined as the lowest concentration of the antimicrobial agent tested that prevented any discernible growth after 24 h.

## Results

**Figure 2:**
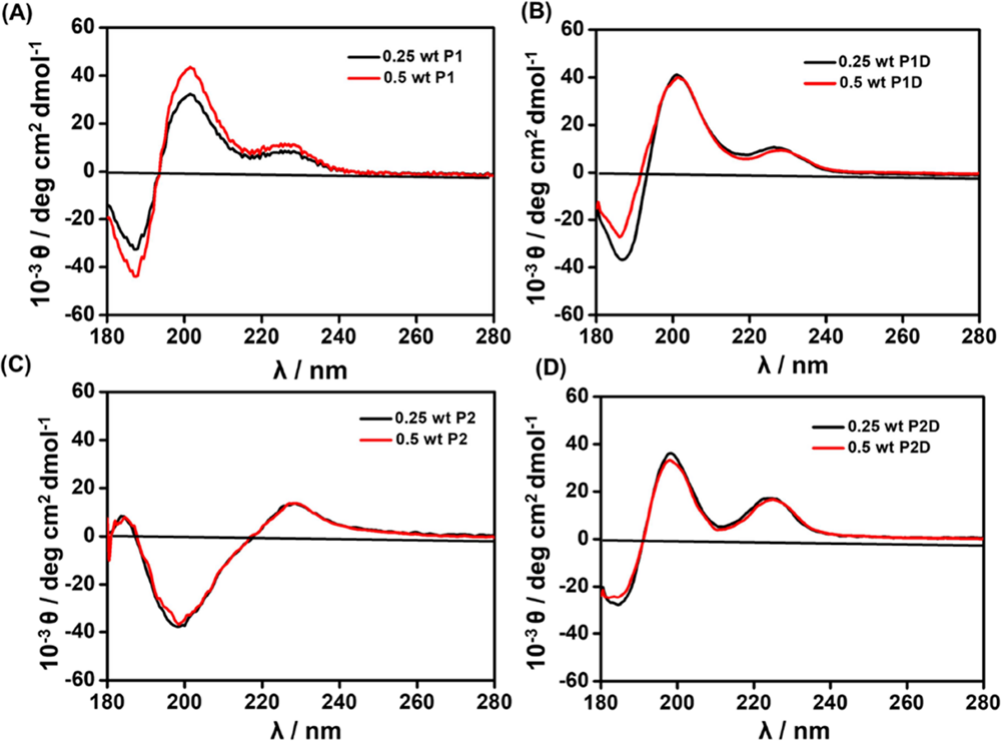
CD spectra of (A) **P1**, (B) **P1D**, (C) **P2,** and (D) **P2D** in 0.25 and 0.5 wt % aqueous solutions.

The four lipopeptides C_16_-YKK abbreviated as **P1**, analogue C_16_-Ykk abbreviated as **P1D**, C_16_-WKK abbreviated as **P2**, and its d-lysine analogue C_16_-Wkk abbreviated as **P2D** were synthesized by solid-phase peptide synthesis methods. Rink amide resin was used as a solid support, and all the protected amino acids were coupled through an amide bond with HBTU and DIPEA as coupling agents. Piperidine was used for Fmoc group deprotection. After the synthesis of the peptides, they were cleaved from the resin by TFA, precipitated in ice-cold diethyl ether, and characterized by ESI-MS, and their purity was checked by reverse-phase HPLC. The characterization data are shown in SI Figures S1–S8. We first examined the peptide conformation using spectroscopic methods. CD spectra are shown in Figure 2. In the CD spectra, the 0.25 and 0.5 wt % aqueous solutions of **P1** displayed a minimum with a negative band at 190 nm and positive bands near 200 nm and 225–230 nm, which suggests unordered and/or extended (polyproline II-like) coil conformation^45−48^ (Figure 2A), and similar features are seen for **P1D** and **P2D** (Figure 2B,D). The spectrum for **P2** in Figure 2C features a negative minimum at 200 nm rather than a positive maximum, although the secondary maximum at 225–230 nm is still present. The band in the CD spectra in the range of 225–230 nm is due to the aromatic amino acid chromophore, i.e., tyrosine for **P1** and **P1D** and tryptophan for **P2** and **P2D**.^45,49,50^

**Figure 3:**
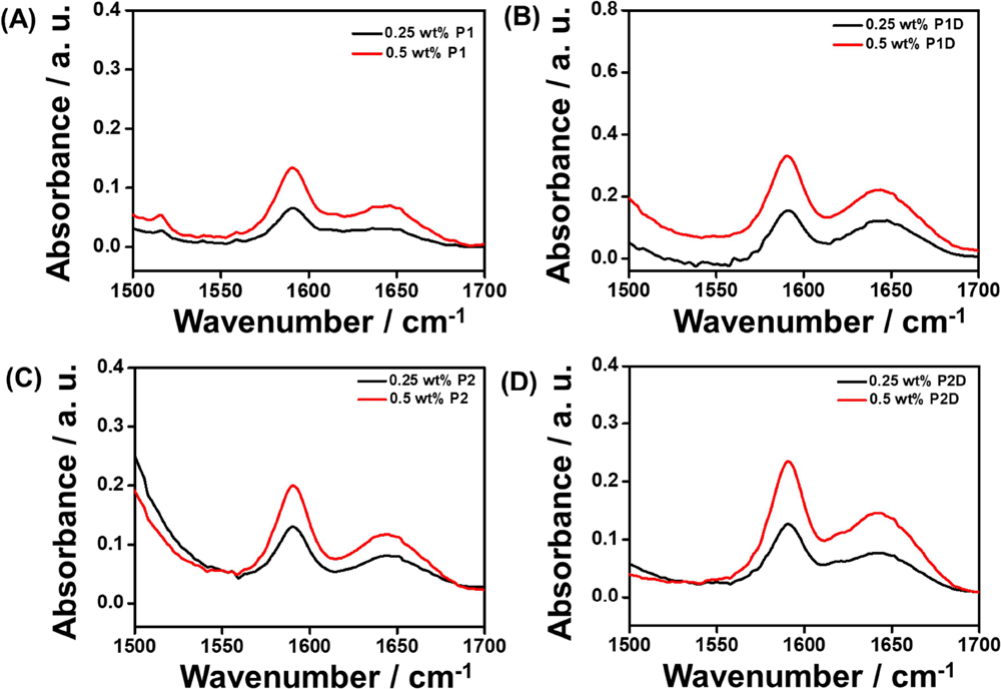
FTIR spectra of (A) **P1**, (B) **P1D**, (C) **P2,** and (D) **P2D** in 0.25 and 0.5 wt % aqueous solutions, respectively.

Next, FTIR spectra were measured. Spectra covering the amide I′ and II′ regions are shown in Figure 3, the amide I region being important to characterize the secondary structure of peptides. From Figure 3, bands in the range of 1500–1700 cm^–1^ were observed for **P1**, **P2**, and **P2D**, which suggest the formation of disordered conformation.^51−53^ For all the lipopeptides, a broad peak in the amide II′ region centered around 1590–1600 cm^–1^ was observed, which can be assigned to aromatic side chain bands.^53^ The presence of the palmitoyl chain in each lipopeptide can be confirmed by the appearance of the peaks corresponding to the CH/CH_2_ /CH_3_ stretching modes of the lipid chains, showing bands centered at ∼2850 and ∼2920 cm^–1^ in the FTIR spectra (Figure S9).^54^

**Figure 4:**
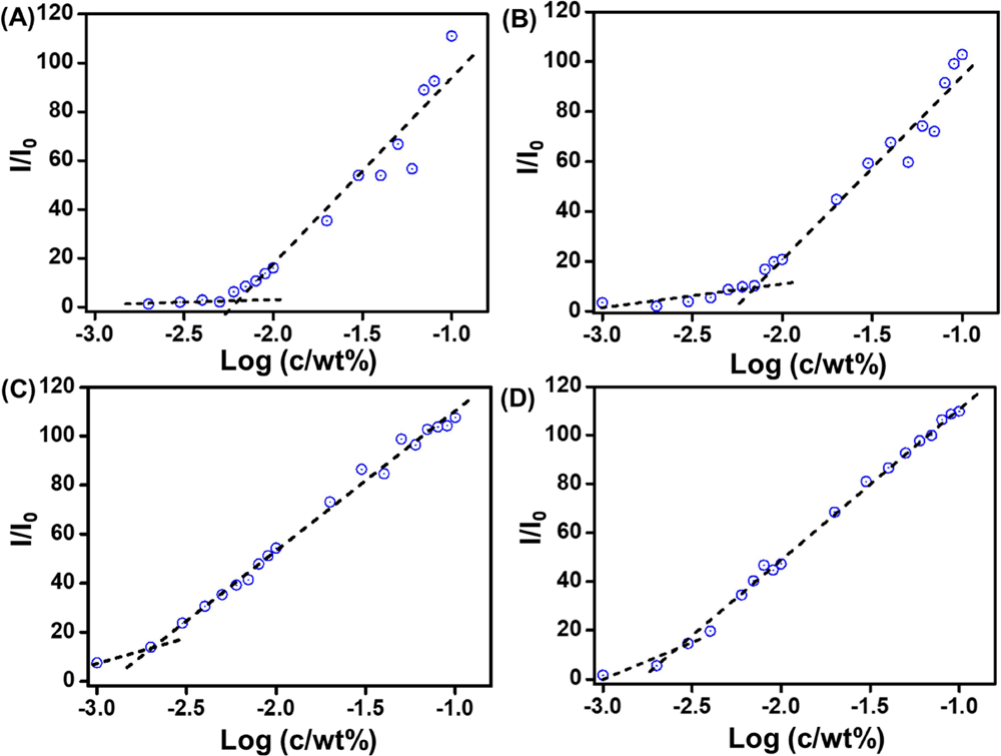
CAC assay using ANS fluorescence peak intensity to determine the CAC value for (A) **P1**, (B) **P1D**, (C) **P2**, and (D) **P2D**.

To determine CAC values of these lipopeptides, fluorescence probe assays using ANS were performed.^43^ Emission spectra of the four lipopeptides were collected at different concentrations after excitation at 356 nm. At higher concentrations, an emission peak at 486 nm is visible. By plotting the fluorescence intensity (*I*/*I*_0_) at 486 nm as a function of the concentration (Figure S10), the concentration at the breakpoint (corresponding to the CAC) was found to be 0.00645 ± 0.05 wt % for **P1** (Figure 4A), 0.00707 ± 0.03 wt % for **P1D** (Figure 4B), 0.00204 ± 0.04 wt % for **P2** (Figure 4C), and 0.00257 ± 0.05 wt % for **P2D** (Figure 4D). The lower CAC values for **P2** and **P2D** are due to the higher hydrophobicity of tryptophan than tyrosine.

**Figure 5:**
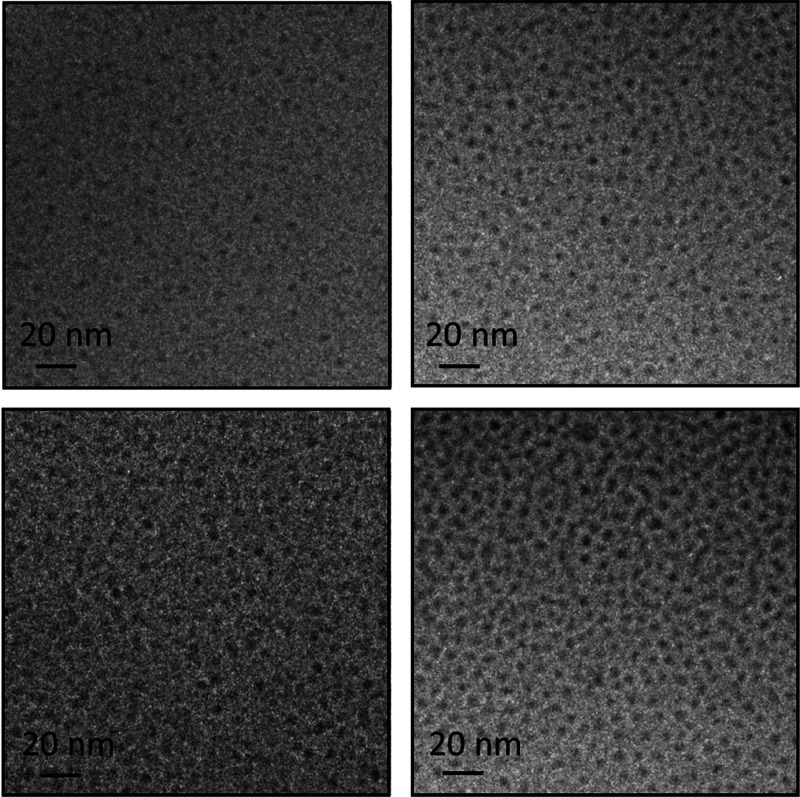
Cryo-TEM images from 1 wt % solutions (A) **P1**, (B) **P1D**, (C) **P2**, and (D) **P2D**.

To investigate the self-assembled nanostructure of these peptides, cryo-TEM imaging was performed for 1 wt % solutions, above the measured CAC values. The images shown in Figure 5 show small spherical micelles for all samples. The mean diameter of the micelles for all four lipopeptides is in the range of 4–9 nm.

**Figure 6:**
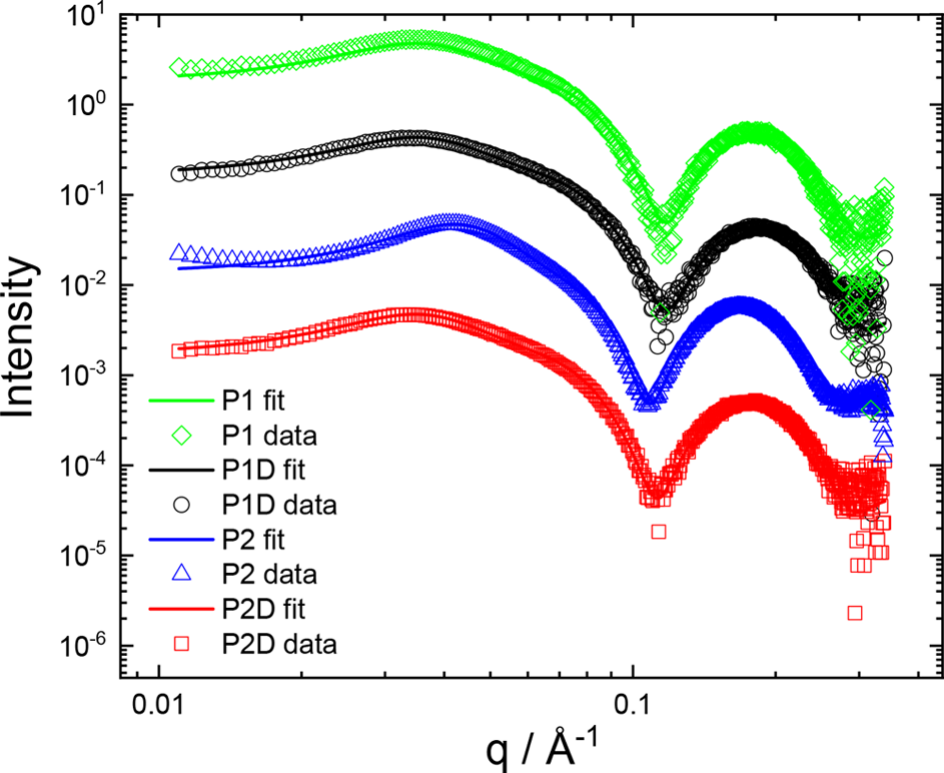
SAXS data for 1 wt % aqueous solution of the four lipopeptides. Open symbols represent measured data (every 5th data point shown for convenience), and solid lines are fitted intensity profiles considering both form and structure factors (fit parameters listed in SI Table S1). Data sets are offset vertically for ease of visualization.

**Figure 7:**
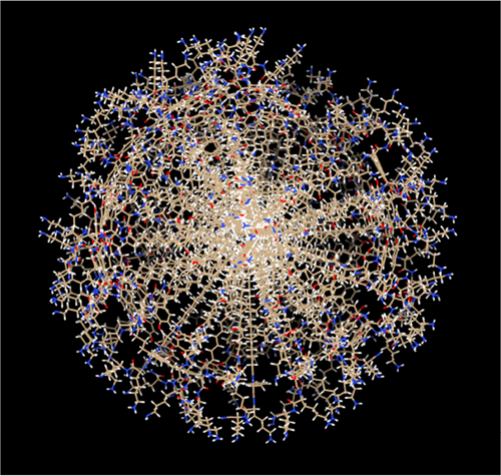
Model of a micelle of **P1** based on SAXS fit parameters.

More quantitative information about these self-assembled nanostructures was obtained from SAXS, also performed for 1 wt % aqueous solutions of the four lipopeptides. Measured SAXS data along with fitted intensity profiles are shown in Figure 6. Consistent with cryo-TEM, the form factors for **P1**, **P1D**, **P2**, and **P2D** can be fitted based on the core–shell sphere structure corresponding to a hydrophobic core and peptide corona. The fit parameters listed in SI Table S1 show outer radii in the range (2.47–2.90 ± 0.4) nm, consistent with cryo-TEM. The inner core radius is 1.45–1.56 nm, which is consistent with the length of an extended C_16_ lipid chain plus the first hydrophobic amino acid (Y or W). The resulting shell thicknesses are in the range of 1.02–1.34 nm, the value for **P2** being significantly larger than for the other three lipopeptides. The shell thickness may be compared to the length of a tripeptide in an extended structure (such as PPII), 3 × 0.31 nm = 0.93 nm (residue translation value taken from ref (55)) and is in good agreement with this value for **P1**, **P1D**, and **P2D**. For **P2**, the shell thickness from SAXS data fitting is larger, suggesting a more extended conformation or a greater hydration layer. The form factor parameters are the same within uncertainties for the other three lipopeptides. The association number can be estimated for **P1** (similar values are expected for **P1D** and **P2D** since the micelle radius and molecular volume are similar) using the volume of a micelle *V*_mic_ = 4/3π(2.52 nm)^3^ = 67.0 nm^3^, while the molecular volume *V*_mol_ = 0.62 nm^3^ was obtained from the hydrophobicity surface/molecular volume calculation in UCSF Chimera.^56^ This leads to an estimated association number *p* = *V*_mic_/*V*_mol_ = (108 ± 20). A schematic showing a micelle of **P1** with this association number is presented in Figure 7.

As well as a contribution due to form factor, it was necessary to allow for structure factor effects in the SAXS data, which produced the broad peak near *q* = 0.04 Å^–1^ evident for all four samples in the data in Figure 6. The simplest structure factor, that for hard spheres, is able to account for this feature, and the fit parameters are included in SI Table S1. The hard sphere radius is *R*_HS_ = 7.9 nm with an effective volume fraction ϕ_p_ = 0.15 for **P1**, **P1D,** and **P2D;** however, the fit parameters for **P2** differ with a smaller hard sphere radius *R*_HS_ = 6.7 nm and larger ϕ_p_ = 0.21. The origin of this difference is at present unclear, but it indicates a difference in intermolecular interactions which may be due to conformational differences, indicated by the CD spectrum in Figure 2C compared with that for the other three lipopeptides and the SAXS form factor analysis just discussed. Peptide WKK seems to be more extended than the other sequences, leading to micelles with a larger shell thickness (although with a lower effective hard sphere radius and larger volume fraction perhaps due to the expanded micelle corona occupying more of the available volume).

**Figure 8:**
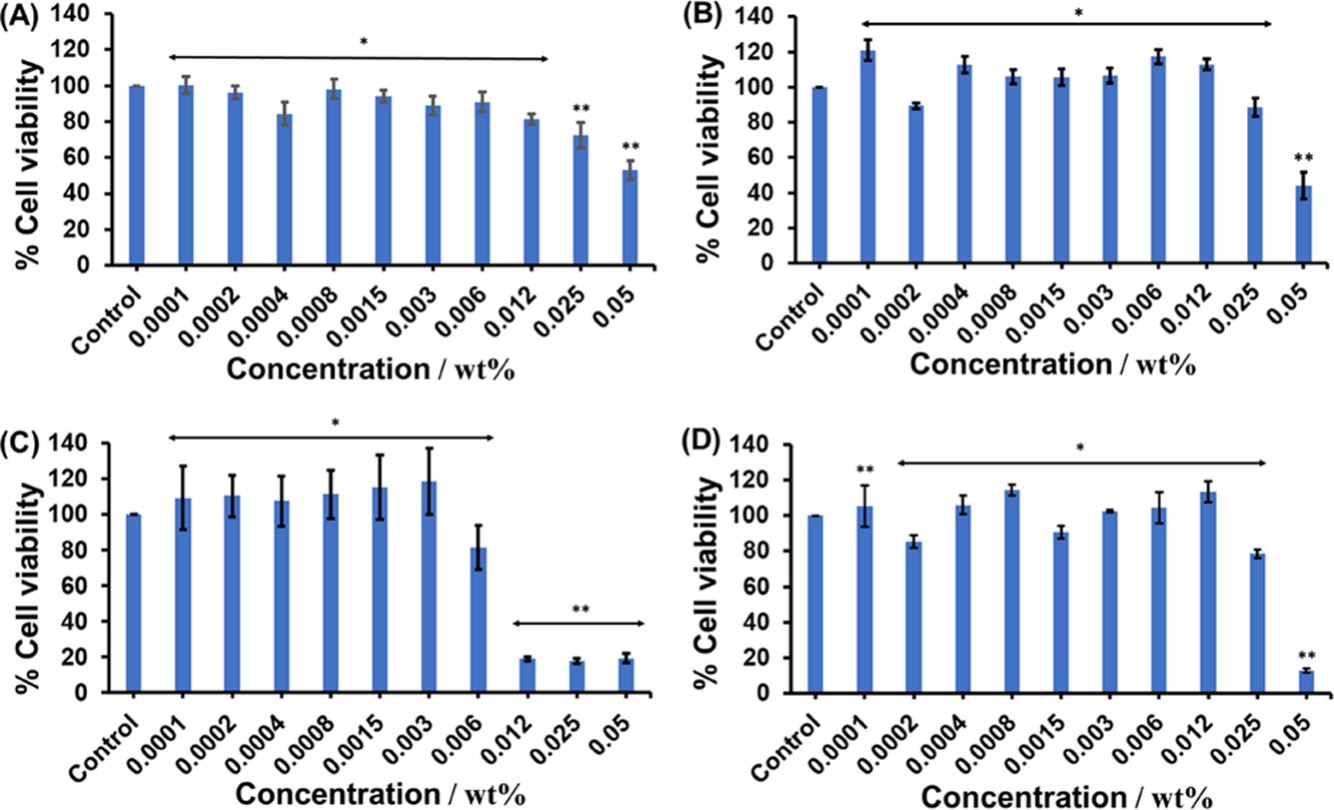
Cell viability data for **P1** (A), **P1D** (B), **P2** (C), and **P2D** (D). The error bar corresponds to the standard deviation of the value from the mean (*n* = 3, *p < 0.05, **p < 0.01 by performing a two-tailed Student’s *t*-test).

To be useful for potential application in vivo, for example, as antimicrobial materials, bioactive molecules must have selective activity and should not be cytotoxic to mammalian cells. Here, the cytotoxicity of all the lipopeptides was investigated by MTT assays on L929 murine fibroblast cell lines. This is an assay of cell metabolic activity. For all the lipopeptides, the cytotoxicity results were obtained after 48 h of cell culture. The data in Figure 8 show that the cell viability is very high (there was no significant difference when compared with control cells) at the lower lipopeptide concentrations for all of the lipopeptides. However, considering concentrations (0.05 wt %) above the CAC for all samples, cell viabilities are as follows: for **P1** 53.1 ± 5.1% (Figure 8A), for **P1D** 44.1 ± 7.6% (Figure 8B), for **P2** 20.2 ± 2.8% (Figure 8C), and for **P2D** 13.0 ± 1.1% (Figure 8D), respectively. These results suggest that self-assembled aggregates are not well tolerated, whereas monomers are completely noncytotoxic.

Since all the molecules are biocompatible at low concentrations, their antibacterial efficacy was examined in preliminary tests using a broth microdilution assay against both Gram-negative and Gram-positive bacteria. Here, *E. coli* K-12, *S. enterica* NCTC 5188 were cultured as Gram-negative bacteria and *S. aureus* NCDO 949 was used as a model Gram-positive bacterium. The lipopeptides were dissolved in water, filter-sterilized, and further diluted in Mueller-Hinton broth, which was used as the growth medium for the experiment to determine MIC values. All four lipopeptides showed antibacterial activity against the tested strains. *E. coli* was most susceptible to compounds **P1** and **P1D** with MICs falling within the range of 15.63–31.25 μg/mL (Table 1). The highest MIC determined was for compound **P2** against *S. enterica*, being within the range of 62.5–250 μg/mL, and all the other MIC determinations are within the range of 31.25–62.5 μg/mL. Overall, the **P1** analogues have lower MIC values than those for the **P2** analogues, especially for *S. enterica*. The lower range MIC value for *E. coli* for **P1**, **P1D**, and **P2** equates to a concentration of approximately 22–23 μM. This is somewhat higher than values reported for these and other bacteria by Makovitzki et al.^38^ for C_16_-KKK and C_16_-KkK, although it is comparable to their values for C_16_-KGK and C_16_-KLK (for Gram-positive, lower for Gram-negative) and is lower than MIC values for most bacterial species for C_16_-KAK, C_16_-KLK and C_16_-Kk and C_16_-K. The values in Table 1 also compare favorably, in general, with those reported by Laverty et al. for C_n_-OOWW lipopeptides, although the latter do seem more active against *Staphylococcus* strains for longer chain homologues.^37^

**Table 1:** Antibacterial Susceptibility Assay Showing MIC Values of the Lipopeptides against Gram-Negative Bacteria *E. coli* K-12, *S. enterica* NCTC 5188, and Gram-Positive Bacteria *S. aureus* NCDO 949 after 24 h Incubation Time

lipopeptides (μg/mL)	*E. coli* K-12	*S. enterica* NCTC-5188	*S. aureus* NCDO 949
**P1**	15.63–31.25	31.25–62.5	31.25–62.5
**P1D**	15.63–31.25	31.25	31.25
**P2**	15.63–62.5	62.5–250	31.25–62.5
**P2D**	31.25–62.5	62.5	31.25–62.5

## Discussion and Conclusions

In summary, all four lipopeptides form spherical micelles, which present lysine residues with high cationic charge at the surface and have good cytocompatibility (below the CAC and in some cases above it) and antimicrobial activity. The lipopeptides form spherical micelles with a similar compact size (radius 2.5–2.9 nm), based on a hydrophobic lipid interior and a shell of unordered/extended peptides (based on conformational analysis from CD and FTIR). Peptide **P2** exhibits some unexpected conformational differences compared with the other three lipopeptides, with a distinct CD spectrum and differences in the SAXS form factor and structure factor parameters which indicate a larger peptide corona in the micelles as well as smaller effective hard sphere radius. It may be noted that cation−π interactions are expected for our lipopeptides due to interactions between the aromatic residues and lysines. In particular, this has been modeled for tryptophan (with indole π system) interacting with lysine (cationic), and it could be a factor in the distinct behavior of C_16_-WKK (**P2**) although this cannot be the full explanation because **P2D** does not exhibit distinct behavior. There will, however, be a complex interplay between cation−π, electrostatic, and conformational (chirality) effects. For all four lipopeptides, the CAC values were obtained from fluorescence probe measurements using ANS, which exhibits enhanced fluorescence in local hydrophobic environments.

All four lipopeptides are cytocompatible with the murine fibroblasts employed at concentrations below the CAC. They also show antimicrobial activity against the Gram-negative and Gram-positive bacteria studied in the preliminary assays presented. In the antimicrobial tests, we used three different bacterial organisms. The first was the Gram-negative bacterium *E. coli*, which is a commensal microorganism found in the intestinal tract of humans and other warm-blooded animals which also includes pathogenic strains including O157:H7. The second was *S. enteritidis* NCTC 5188, also a Gram-negative bacterium that is one of the most well-known foodborne pathogens, and the third was the Gram-positive bacterium *S. aureus* that is also a commensal organism residing in the skin of humans and other animals and is also a human pathogen that can cause a wide range of diseases ranging from foodborne illness to nosocomial infections.

All compounds tested exerted antimicrobial activity against both Gram-negative bacteria *E. coli* K-12, *S. enteritidis* NCTC 5188, and against the Gram-positive bacterium *S. aureus* NCDO 949. This clearly indicates that these compounds have a relatively broad range of antimicrobial activity, although further work on a wider range of organisms would be required to establish this pattern. *E. coli* K-12 was relatively more susceptible to compounds **P1** and **P1D**. However, no major differences were observed between the compounds and the microorganisms tested, apart from the increased variability between replicates for compound **P2**.

From the results in Figure 8, it was observed that the concentration at which the lipopeptides show toxicity to L929 fibroblasts is above 0.006 wt %, i.e., 0.06 mg/mL. The lower MIC values of these lipopeptides for antimicrobial studies are around 0.015–0.03 mg/mL. In this concentration range, the lipopeptides are completely noncytotoxic. Thus, these lipopeptides are promising for future in vivo studies. Here, MIC values are found in the ten’s of micromolar range, which compares well with previous results, although these are higher than values found for lipopeptides containing more cationic lysine residues (C_16_-KKK and C_16_-KkK).^38^ Thus, a larger number of lysine residues can enhance antimicrobial activity, although this must be balanced against reduced cytocompatibility, for instance, C_16_-KKK shows the highest hemolysis among all conjugates studied by Makovitzki et al.^38^ The comparable range of MIC values for our lipopeptides compared with those from tripeptide conjugates containing two lysines in this report is also promising for future in vivo applications, already demonstrated by these authors via a murine model of fungal infection. Our obtained MIC values are higher than those for nonpeptide antimicrobial pharmaceutical molecules in clinical practice, with MIC50 values of 0.04–4 μg/mL for *E. coli*, for example.^57^ However, they are lower than those reported for the clinically relevant cyclic lipopeptide polymyxins including colistin against *S. aureus*, but not Gram-negative bacteria.^58,59^ Our MIC values are comparable to those reported for some AMPs under development for clinical application.^60^ It is notable that our lipopeptides are active against more challenging Gram-negative bacteria (with an additional outer cell membrane that Gram-positive bacteria lack). This is consistent with the proposed mode of action of cationic AMPs in disrupting bacterial cell membranes which are enriched in anionic lipids compared with mammalian cells, rich in zwitterionic lipids.^61^

Future work should extend the preliminary in vitro antimicrobial tests reported here, to in vivo studies, also the mode of action could be investigated. The development of resistance as well as activity against biofilms could be examined, as assessed, for example, in our study on surfactant-like peptides that contain arginine,^62^ rather than lysine as cationic residue as in the present work. There is scope to further improve the activity of our AMPs by a combination therapy approach, as already used with antimicrobial agents in clinical practice. Since our initial results show promise, we also plan in the future to examine activity against other bacteria and to further probe biocompatibility via hemolysis assays.

## Supporting Information Available

HPLC and MS data for lipopeptides, FTIR spectra, fluorescence spectra from CAC assay, and SAXS data fit parameters (PDF)
