# Supplementary material for: Self-Assembly and Antimicrobial Activity of Lipopeptides Containing Lysine-Rich Tripeptides
Source: Biomacromolecules. 2024 Jan 11;25(2):1205–13. doi: 10.1021/acs.biomac.3c01184 (PMC10865344; doi:10.1021/acs.biomac.3c01184)
Supplement: Supplementary file 1 — bm3c01184_si_001.pdf [file bm3c01184_si_001.pdf]

Supporting Information

Self-Assembly and Antimicrobial Activity of Lipopeptides Containing Lysine-Rich Tripeptides

Anindyasundar Adak,[a] Valeria Castelletto,[a] Ana de Sousa,[a] Kimon-Andreas Karatzas,[a] Callum Wilkinson,[a] Nikul Khunti,[b] Jani Seitsonen[c] and Ian W. Hamley*[a]

[a] *School of Chemistry, Pharmacy and Food Biosciences, University of Reading, Whiteknights, Reading RG6 6AH, U.K.*

[b] *Diamond Light Source, Harwell Science and Innovation Campus, Chilton, Didcot OX11 0DE, U.K.*

[c] *Nanomicroscopy Center, Aalto University, Puumiehenkuja 2, FIN-02150 Espoo, Finland*

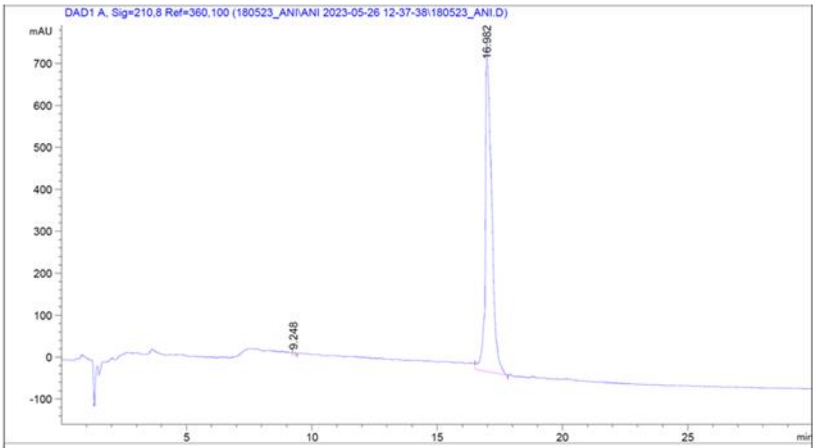

Peak #	RetTime [min]	Sig	Type	Area [mAU*s]	Height [mAU]	Area %
1	9.248	1	VV	52.16250	5.65082	0.3812
2	16.982	1	VV	1.36299e4	782.20624	99.6188
Totals :				1.36821e4	787.85706	

Figure S1. HPLC chromatogram of **P1**.

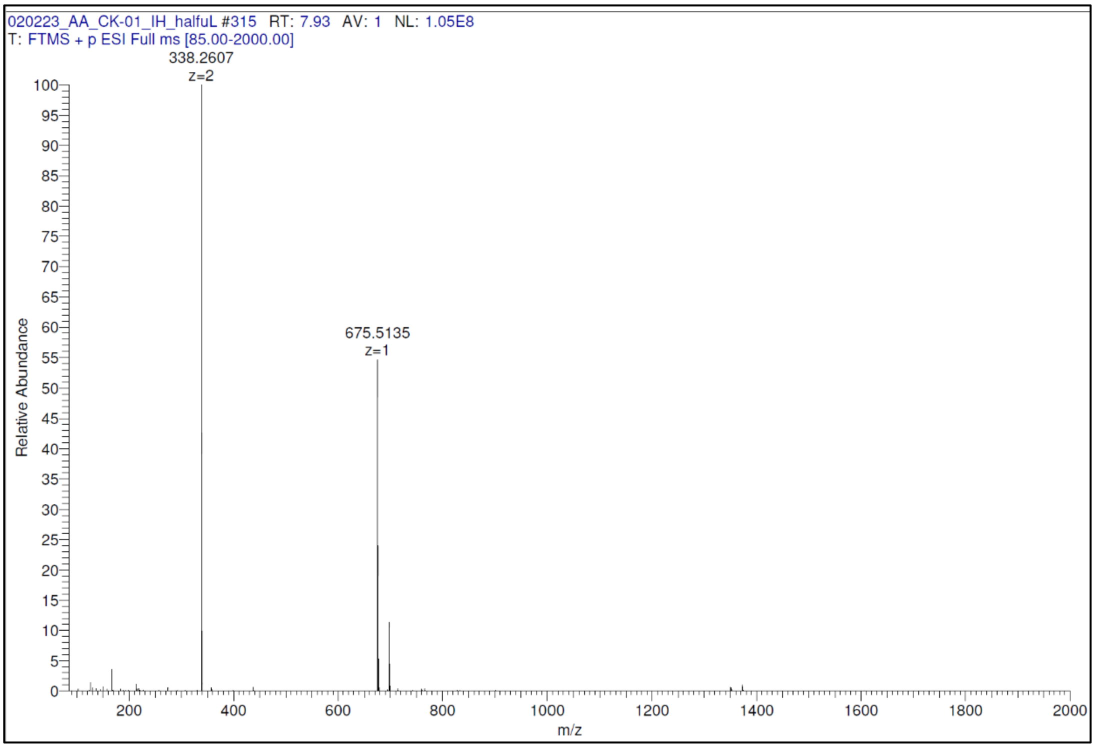

Figure S2. ESI-MS data of **P1** (M = 675.5, M/2 = 338.2).

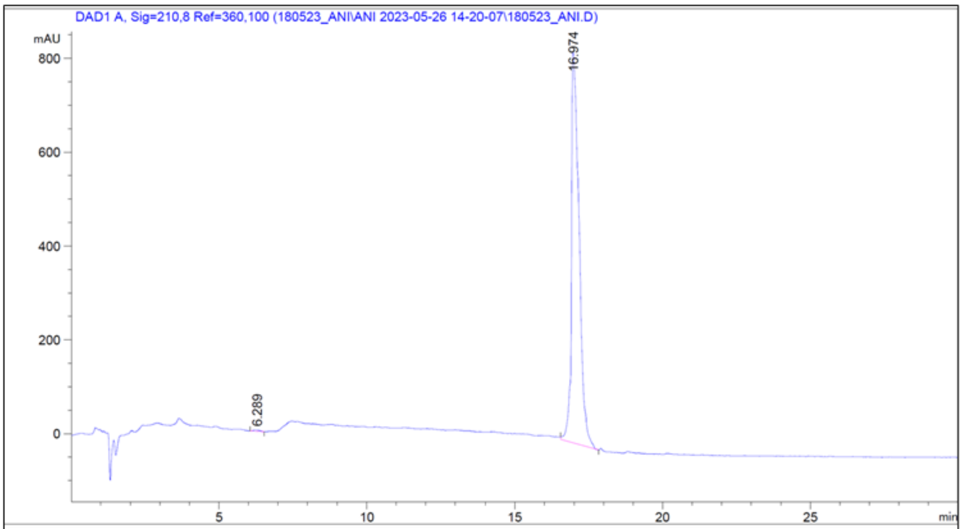

Peak #	RetTime [min]	Sig	Type	Area [mAU*s]	Height [mAU]	Area %
1	6.289	1	VV	45.54868	3.24688	0.3034
2	16.974	1	VV	1.49669e4	831.77246	99.6966
Totals :				1.50125e4	835.01934	

Figure S3. HPLC chromatogram of **P1D**.

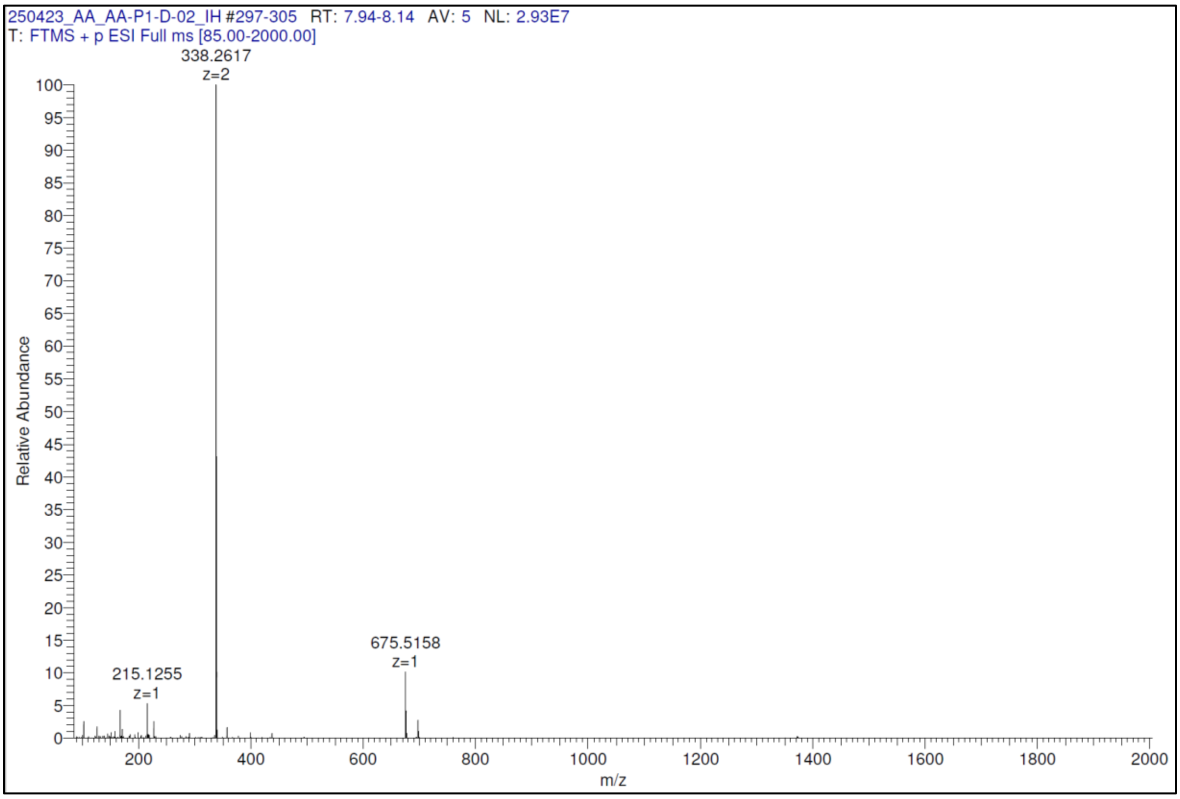

Figure S4. ESI-MS data of **P1D** (M = 675.5, M/2 = 338.2).

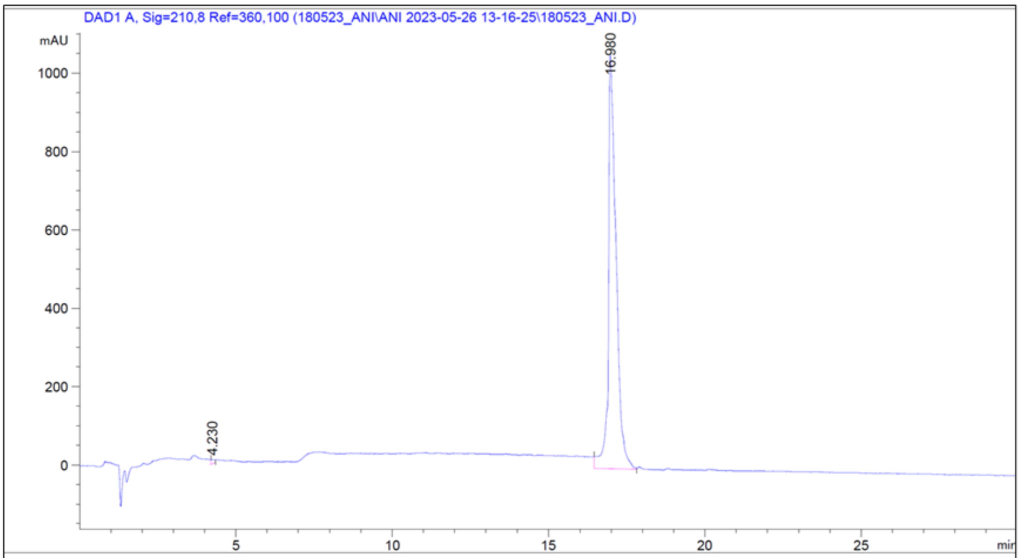

Peak #	RetTime [min]	Sig	Type	Area [mAU*s]	Height [mAU]	Area %
1	4.230	1	VB	91.64943	11.64650	0.5072
2	16.980	1	BV	1.79794e4	1055.65784	99.4928
Totals :				1.80710e4	1067.30434	

Figure S5. HPLC chromatogram of **P2**.

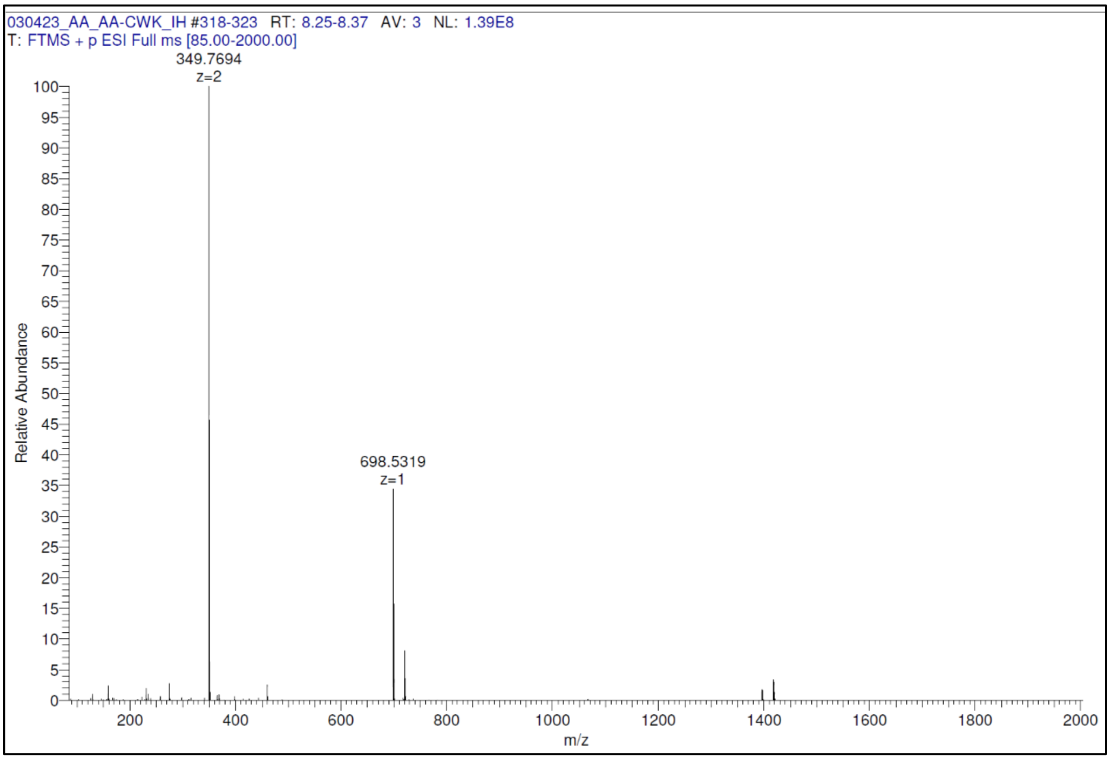

Figure S6. ESI-MS data of **P2** (M = 698.5, M/2 = 349.7).

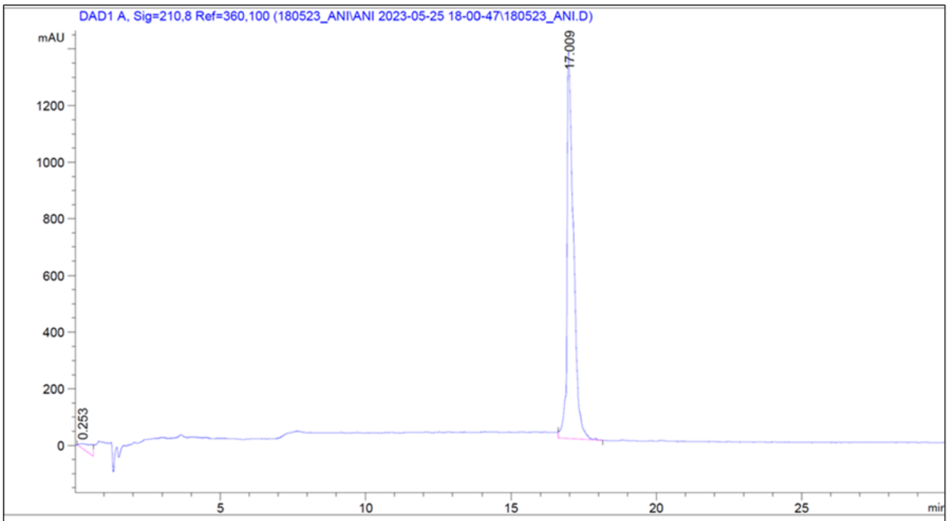

Peak #	RetTime [min]	Sig	Type	Area [mAU*s]	Height [mAU]	Area %
1	0.253	1	BB	723.58441	16.84625	3.3110
2	17.009	1	BB	2.11301e4	1309.92188	96.6890
Totals :				2.18537e4	1326.76813	

Figure S7. HPLC chromatogram of **P2D**.

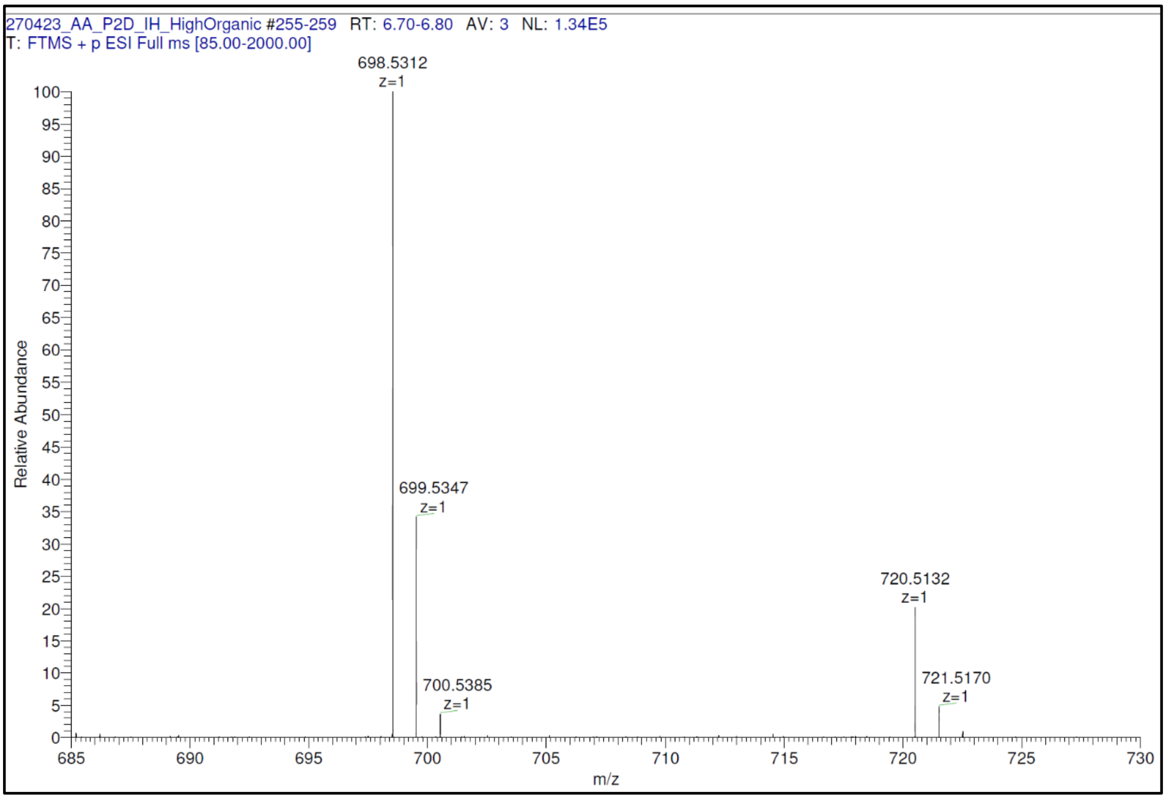

Figure S8. ESI-MS data of **P2D** (M = 698.5, M+Na^{+} = 720.5).

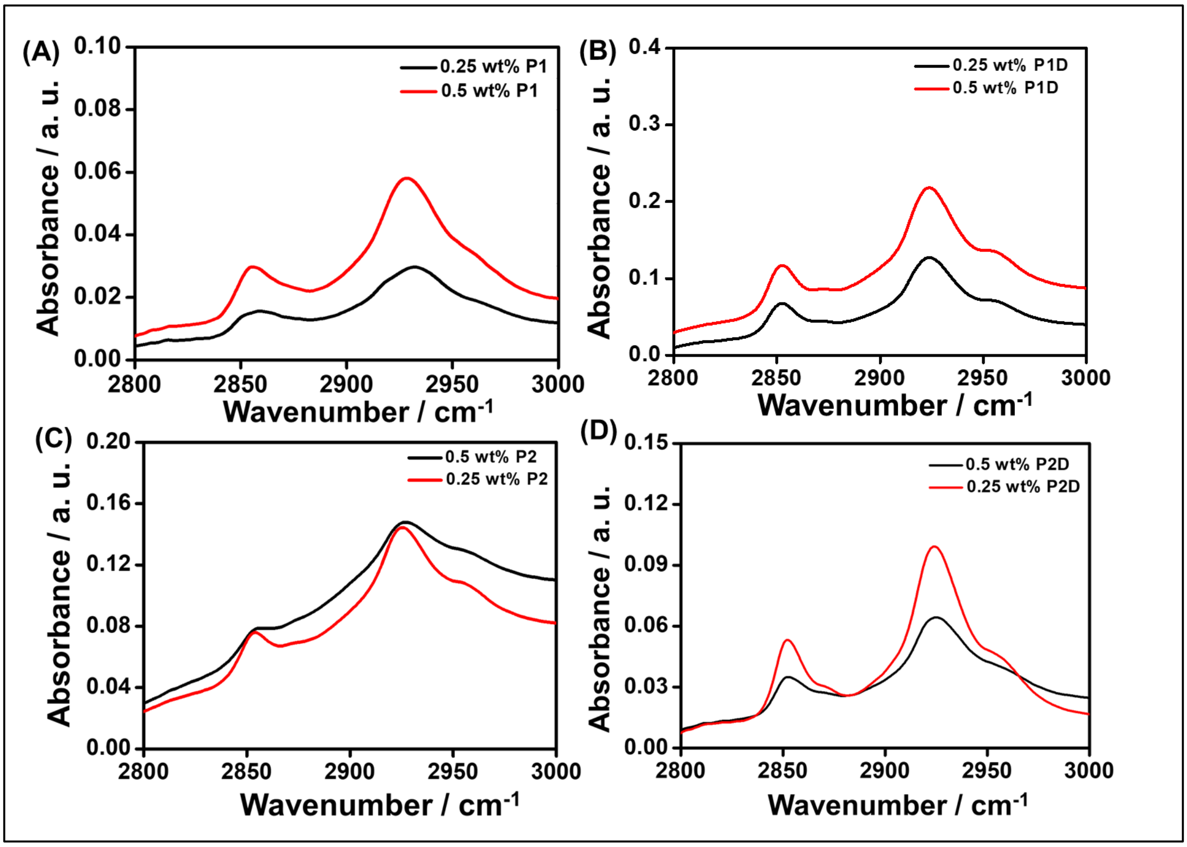

Figure S9. Alkyl chain vibrational band region of FTIR spectra of (A) **P1**, (B) **P1D**, (C) **P2**, and (D) **P2D** in 0.25 wt% and 0.5 wt% aqueous solutions respectively.

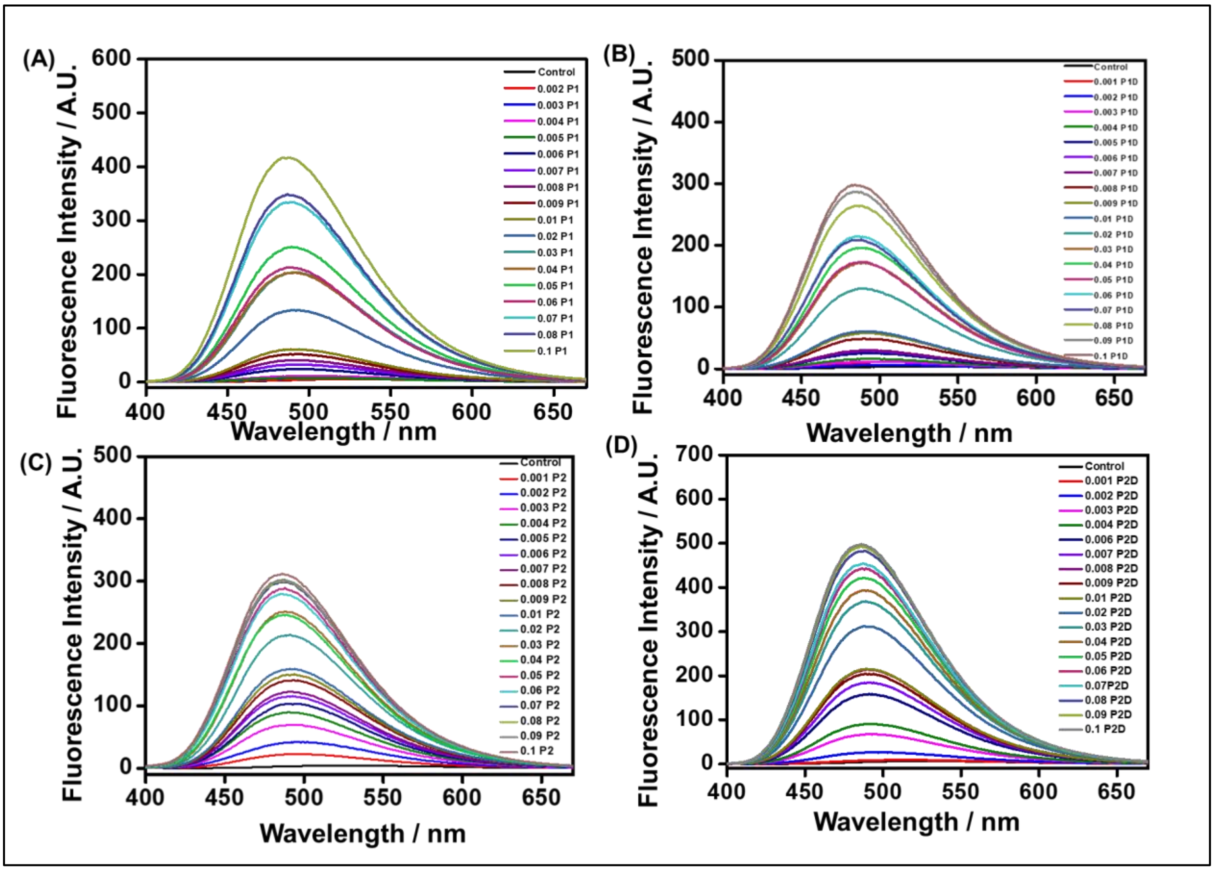

Figure S10. CAC study by ANS: Fluorescence curves of ANS containing various concentrations (wt%) (A) **P1**, (B) **P1D**, (C) **P2**, (D) **P2D**.

Table S1. SAXS data fitted parameters. SAXS data fitted using SASfit,[1-2] using a core-shell sphere form factor model and hard sphere structure factor. All data for 1 wt% samples at native pH = 4.

**Parameter**	**P1**	**P1D**[a]	**P2**	**P2D**
R_o / Å	25.2	24.7	29.0	26.3
ΔR / Å	4.4	4.6	3.8	4.4
R_i / Å	14.5	14.5	15.6	15.1
μ	-1.30	-1.30	-1.39	-1.32
η / cm^{-1}	5.35×10^{-6}	5.35×10^{-6}	5.35×10^{-6}	5.36×10^{-6}
R_{HS} / Å	78.5	78.5	66.5	79.3
ϕ_p	0.15	0.15	0.21	0.16
BG / cm^{-1}	4.0×10^{-4}	4.0×10^{-4}	7.1×10^{-4}	4.0×10^{-4}

Parameters: **Form Factor** – R_o, outer radius (Gaussian polydispersity ΔR); R_i, inner core radius; μ, scattering contrast of inner core (relative to shell); η, scattering contrast of shell; **Structure Factor** – R_{HS}, hard sphere radius; ϕ_p volume fraction; **Background** – BG

[a] Parameters as for **P1** except R_o and ΔR.

References

(1) Bressler, I.; Kohlbrecher, J.; Thünemann, A. F., SASfit: a tool for small-angle scattering data analysis using a library of analytical expressions. *J. Appl. Cryst.* **2015,** *48*, 1587-1598.

(2) Kohlbrecher, J.; Bressler, I., Updates in SASfit for fitting analytical expressions and numerical models to small-angle scattering patterns. *J. Appl. Cryst.* **2022,** *55*, 1677-1688.
